# Inhibiting Fatty Acid Amide Hydrolase Ameliorates Enteropathy in Diabetic Mice: A Cannabinoid 1 Receptor Mediated Mechanism

**DOI:** 10.3390/vetsci9070364

**Published:** 2022-07-16

**Authors:** Vikram Thakur, Mohammad Bashashati, Josue Enriquez, Munmun Chattopadhyay

**Affiliations:** 1 Center of Emphasis in Diabetes and Metabolism, Department of Molecular and Translational Medicine, Texas Tech University Health Sciences Center El Paso, El Paso, TX 79905, USA; vikram.thakur@ttuhsc.edu; 2 Division of Gastroenterology, Department of Internal Medicine, Texas Tech University Health Sciences Center El Paso, El Paso, TX 79905, USA; mohammad.bashashati@ttuhsc.edu; 3 Graduate School of Biomedical Sciences, Texas Tech University Health Sciences Center El Paso, El Paso, TX 79905, USA; josue.enriquez@ttuhsc.edu

**Keywords:** diabetes, gastrointestinal, endocannabinoid, fatty acid amide hydrolase

## Abstract

**Simple Summary:**

Gastrointestinal (GI) disorder is a debilitating complication of diabetes that interferes with the quality of life of the patients and current medical treatments are not very effective. High blood glucose levels in diabetics and the duration of diabetes could lead to complications of the GI tract, such as enteropathy and gastroparesis. Enteropathy is a disorder of the GI tract and can manifest as diarrhea, abdominal pain, constipation, and fecal incontinence. About 20% of patients affected with long-standing diabetes are at risk of diarrhea or fecal incontinence. This study was focused on assessing specific GI symptoms as well as manifestations in Type 1 diabetic mice and evaluated the treatment options for this distressing condition.

**Abstract:**

Gastrointestinal (GI) dysmotility in diabetics exhibits fecal incontinence or constipation which affects patients’ quality of life. In this study, we aimed to understand the pattern of GI transit in type 1 diabetic (T1D) mice and whether inhibiting endocannabinoid degradation would exhibit therapeutic effect. Whole gut-transit time and fecal-pellet output were measured at 16 week post-diabetes. T1D mice treated with fatty acid amide hydrolase (FAAH) inhibitor URB597 showed reduced fecal output as well as improved gut transit time. Cannabinoid 1 receptor antagonist, AM251 blocked the effects of URB597, which may demonstrate that FAAH inhibitor is a potential remedial strategy for GI dysmotility.

## 1. Introduction

Diabetes is a significant public health problem that affects the global population and is heading towards an epidemic. According to a report by the American Diabetes Association, around 30.5 million people in the United States, or about 9% of the total population, have diabetes and approximately 1.25 million of them have type 1 diabetes (T1D). The rising rate of diabetes has also highlighted complications associated with diabetes that affect the gastrointestinal tract. Chronic hyperglycemia and the duration of diabetes could lead to complications of the gastrointestinal tract, such as enteropathy and gastroparesis. It has been reported that gastrointestinal symptoms affect up to 75% of patients with diabetes [1]. Enteropathy is a less recognized complication of the GI tract and can manifest as diarrhea, abdominal pain, constipation, and fecal incontinence [2]. About 20% of patients affected with longstanding diabetes are at risk of diarrhea or fecal incontinence [3]. Abnormal GI motility, in combination with irregular secretion and absorption leading to a decline in the contractile functional ability of the gut musculature as a result of loss of cells within the enteric nervous system [4,5], has been implicated as the main causal factor for these conditions [6]. Other factors such as mucosal inflammation could also contribute to disorders of the GI tract [7]. Chronic inflammation is known to negatively impact the functioning of the interstitial cells of Cajal (ICC) by affecting the contractile mechanism, altering neurotransmission and sensitizing visceral afferent neurons [8]. Several studies have shown that activation of the endocannabinoid system may impact the development of diabetes and its complications by promoting oxidative stress and inflammation [9]. Diabetes is one of the most common causes of oxidative stress and it is well known that hyperglycemia has a negative effect on gastric emptying [10]. In patients with long term T1D, bowel movements are affected as a result of damage to the nerves that control the musculature in the stomach. This damage could affect the entire GI tract, causing rapid excavation of the GI tract and higher incidence of diarrhea in patients with diabetes. The production of nitric oxide by the nNOS neurons also plays an important role in gut motility by mediating smooth muscle relaxation in the GI tract [11]. The mechanistic pathogenesis of diabetes leading to diarrhea is not fully understood, but autonomic neuropathy [12,13], in conjunction with glucose levels, as well as hormonal and neurotransmitter imbalances in the gut could play important roles in the disturbances in the GI tract as a result of T1D. Genetic predisposition to diabetes could also play a role in the pathogenesis of diabetic enteropathy [3].

The endocannabinoid system is universally present throughout the body and is involved in many physiological functions, including the regulation of gastrointestinal function [14]. For decades, cannabinoids have been shown to be effective in a variety of GI symptoms, including nausea, intestinal pain, and fecal incontinence. Endocannabinoids are biological regulators of intestinal motility [15,16]. The endocannabinoid system acts via at least two types of cannabinoid receptors, CB1 and CB2 receptors, which are present throughout the body. Endogenous agonists for cannabinoid receptors, i.e., anandamide, 2-arachidonoyl glycerol, and 2-arachidonyl glyceryl ether are known as endocannabinoids [17]. CB1 and CB2 receptors are involved in a number of physiological processes including desire to eat, mood stabilization, pain perception, and memory [18,19]. CB receptors are part of the G protein-coupled receptor superfamily and are present in several cell types [20]. Anandamide and 2-arachidonoylglycerol (2-AG) are 2 endocannabinoids that are synthesized as needed from cell membrane arachidonic acid derivatives and stimulate cannabinoid receptors; they have short half-life before being degraded by the enzymes fatty acid amide hydrolase (FAAH) and monoacylglycerol lipase (MAGL), respectively [14,21]. Pharmacological interventions of endocannabinoids have demonstrated therapeutic advancements toward a number of pathological conditions including neurodegenerative and gastrointestinal diseases. Agonists for endocannabinoid receptor decrease gastrointestinal motility [22,23]. Studies have shown that CB1 antagonist alters motility of rodent small intestine, which suggests the presence of CB1 receptors in the enteric system [22,24].

In this study, we evaluated the whole gut transit time in T1D mice. The objective of the current study was to assess the role of the endocannabinoid system in the regulation of gut transit in T1D mice. For this purpose, we used streptozotocin (STZ)-induced T1D mice model and compared diabetic, control, vehicle or treated groups for the total fecal output and the whole gut transit time. The effect of treatments that impede endocannabinoid degradation has not been studied thoroughly in diabetic enteropathy. Therefore, in this study, the diabetic animals were treated with FAAH inhibitor URB597 and also CB1 receptor antagonist AM251 and these animals were evaluated for the fecal output and whole gut transit time post-treatment.

## 2. Methods

### 2.1. Experimental Design

Seven-week-old male C57BL/6J mice (Jackson Laboratories, Bar Harbor, ME, USA) were used in this study. The mice were housed at room temperature under 12 h light/dark cycle in compliance with approved institutional animal care and use protocols. The animals were acclimatized to the animal facility before induction of diabetes. All the animals were humanely euthanized at the end of the study. Experiments were performed in compliance with approved institutional animal care and use protocols (IACUC, TTUHSC El Paso, El Paso, TX, USA). To ensure that the appropriately minimal number of mice was used in each experimental context, we calculated the number of experimental mice by performing sample size estimation with power analysis (>80%) and alpha (0.01) in consultation with institutional biostatistician. Each control, diabetic, or treatment group had 5–8 animals per group.

### 2.2. Diabetes Induction

Two sets of mice (one cohort for fecal output and one cohort for gastric emptying studies) were injected with two intraperitoneal injections of 100 mg/kg of STZ in citrate buffer at an interval of 48 h to induce diabetes [25]. Three days after STZ injections, blood glucose levels were measured from whole blood of tail vein using a One Touch Ultra glucose meter (LifeScan, Inc.; Milpitas, CA, USA). Mice with blood glucose levels ≥300 mg/dL were included in the study. The studies were completed 16 weeks after diabetes.

### 2.3. Fecal Pellet Output Study

Two to three animals were kept in each cage with ad libitum food and water. Food and water were removed 2 h before experiment. Following which, each experimental animal was transferred to a clean individual cage devoid of bedding, water, and food for another 30 min for acclimatization. To avoid possible stress of a new empty cage and handling, fecal pellets were not collected for the first 30 min. Thereafter, pellets were collected every 30 min. This experiment was planned based on our previously published work [26,27], which showed no significant variability within individual group. The number of pellets at 30, 60, 90, and 120 min was counted, collected, and weighed immediately after collection which constituted the wet weight. The pellets were left to dry for 24 h and weighed again, which constituted the dry weight. Five mice from each group were tested.

### 2.4. Drugs

We used URB597, a selective inhibitor of fatty acid amide hydrolase (FAAH), and AM251, a potent CB1 receptor antagonist for treatment. The experimental animals were divided into 5 groups: control-vehicle, control + URB597, diabetic + URB597, diabetic + URB597 + AM251, and diabetic-vehicle. Each group consisted of 5–8 animals per group. All 5 groups were injected either with vehicle or the drugs for the fecal output study. Another set of animals was injected either with vehicle or the drugs followed by gastric gavage with Evans Blue for the whole gut transit study.

AM251 (0.5 mg/kg) [24] was prepared in 20% Tween 80, saline, and 2.5% DMSO and URB597 (5 mg/kg) was prepared in 2.5% DMSO, saline, and 2.5% Tween 80; both were administered intraperitoneally. To understand whether CB1 receptor was involved in the diabetic gastric motility, we used AM251, a CB1 receptor antagonist, followed by URB597 treatment to investigate the effect of FAAH inhibitor. There was no difference between the control and the control + URB597 groups (data shown in Supplementary Figure S2). For the diabetic + URB597 + AM251 group, AM251 was injected while the mice were still in their original cages followed by URB597 injections after 10 min. The mice injected with either URB597 or vehicle were left in their cages for another 15 min. For the whole gut transit study, control, vehicle, and treated animals received Evans Blue gastric gavage, then they were transferred to empty cages.

### 2.5. Evans Blue Experiment

Whole gut transit was measured with intragastric gavage of Evans Blue. Mice were fasted overnight with access to water up to 2 h before experiment. Five mice per group were acclimatized to individual empty cages (without bedding, food/water). The control, diabetic-vehicle, and treated mice (URB597 alone, URB597 + AM251) were then gavaged with 200 μL of 5% Evans Blue suspension in 5% gum Arabic. Mice were monitored for the transit of Evans Blue through the gut. The time of first blue bowel movement was measured in min and was considered to be the whole gut transit time.

### 2.6. Data Analysis

We calculated the number of experimental mice by performing sample size estimation with power analysis (>80%) and alpha (0.01). For fecal output and whole gut transit studies, we assessed 5 mice/group. For the analysis of whole gut transit studies, one-way analysis of variance (ANOVA) were used followed by Bonferroni multiple comparison tests in post hoc analysis. All the statistical analysis was carried out using Graph-Pad and Systat 13 (SPSS, Inc., Chicago, IL, USA). Differences with a *p*-value of <0.05 were considered significant. Data were presented as mean ± SEM.

## 3. Results

### 3.1. Induction of Diabetes Changed Blood Glucose Levels Not Body Weight

Animals injected with STZ showed significant increases in blood glucose levels from 140.2 ± 15.8 to 508.3 ± 20.3 mg/dL (*p* < 0.001), while the blood glucose remained stable in the control animals 146.1 ± 10.4 mg/dL. Diabetic animals did not gain any weight and remained around 26.2 ± 1.6 gm after 16 weeks of diabetes. Weight and blood glucose levels were measured weekly at first, and then monthly (see Supplementary Figure S1). Control animals continued to gain weight as part of their natural growth from 26.1 ± 2.8 gm to 34.2 ± 1.3 gm after 16 weeks (*p* ≤ 0.001). The treatment groups for the control and diabetic animals were randomly selected from each category and, since the treatments were short term, there were no post treatment measures of weight and blood glucose levels.

### 3.2. Increased Output in the Fecal Pellet Was Ameliorated by an FAAH Inhibitor in Diabetic Animals

**Figure 1:**
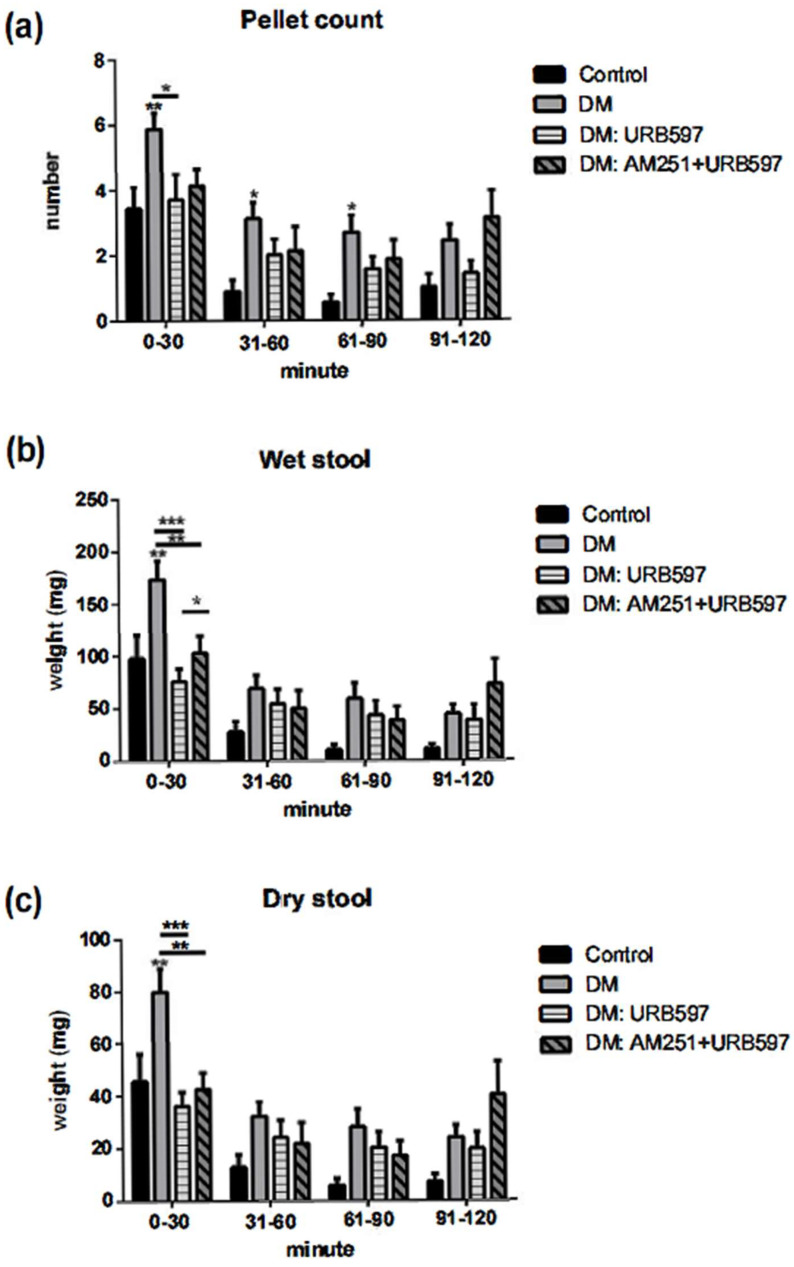
The effect of STZ-induced diabetes and URB597 (5 mg/kg, IP) on fecal output in mice. STZ significantly increased fecal pellet counts as well as wet and dry stool weights (** *p ≤* 0.01 vs. control). URB597 reversed the effects of STZ in the discussed fecal parameters. AM251 (0.5 mg/kg, IP) did not block the effects of URB597. For each group, *n* = 5. Two-way ANOVA: F (3, 144) = 17.20, F (3, 144) = 19.26, and F (3, 144) = 16.16 for row factors in panels (**a**–**c**), respectively (ANOVA *p ≤* 0.001 all panels) (underlined * *p ≤* 0.05, ** *p ≤* 0.01, *** *p ≤* 0.001 vs. DM). DM bar indicates diabetic mice treated with vehicle.

The development of diabetes in mice was associated with a substantial increase in the fecal pellet output as measured after 120 min. The significant increase was observed primarily within the first 90 min of the fecal output measurement. The number of pellets collected after 30 min was 3.4 ± 1.9 for the control group, whereas it was 5.8 ± 1.8 for the diabetic group (*p* ≤ 0.01). The effect of increased fecal output counts in diabetic animals was attenuated by the FAAH inhibitor URB597 (5 mg/kg) treatment after 30 min (Figure 1a). Treatment with URB597 altered the number of fecal pellet count to 3.8 ± 2.0 (*p* ≤ 0.05). There was no difference between control and control + URB597 groups, therefore, the data from the URB597-treated control mice were not included in the graph. Diabetic animals showed substantially increased pellet count as compared with the control animals at 60 and 90 min of fecal pellet collection, whereas treatment with URB597 was effective either at 60 or 90 min after treatment. The pellet count was not significantly different between 90 and 120 min collections.

To understand whether CB1 receptor is involved in the diabetic gastric motility, we used AM251, a CB1 receptor antagonist, followed by URB597 treatment to study the effect of FAAH inhibitor. The data from this experiment suggest that the effect of the FAAH inhibitor on fecal output was not altered by AM251. Treatment with AM251 with URB597 showed no changes in the group treated with only URB597.

### 3.3. Effects of FAAH Inhibitor on Wet and Dry Fecal Pellet Production

The data collected from the fecal output study included counting and weighing of the fecal pellets excreted by individual mice over a period of 2 h and it exhibited a significant increase in the T1D animals as compared with the control counterparts (Figure 1). Our results show that pretreatment of diabetic animals with URB597 eliminated the increased fecal output activity. There was a reduction in the number of wet feces in mice treated with URB597, but the difference was only significant (*p* ≤ 0.001) within 30 min of fecal pellet collection. The wet stool measurement was significantly higher in diabetic animals throughout the experiment as compared with the control animals. By measuring wet and dry stool weight, we aimed to understand whether the increase in fecal pellet count in diabetes was due to the increase in the liquid component of the stool. The treatment with FAAH inhibitor reduced the number of fecal pellets as well as the weight of the wet stool. Pretreatment with CB1 antagonist AM251 partially reversed the effect in the wet stool content, but it was not significantly different in dry stool content that was collected within 30 min of the experiment. Our study results demonstrated that induction of diabetes with STZ increased both wet and dry stool weight to the same extent and more prominently within the first 30 min (Figure 1b,c). Treatment did not change the wet and dry content after 30 min of the experiment.

### 3.4. Whole Gut Transit Time

**Figure 2:**
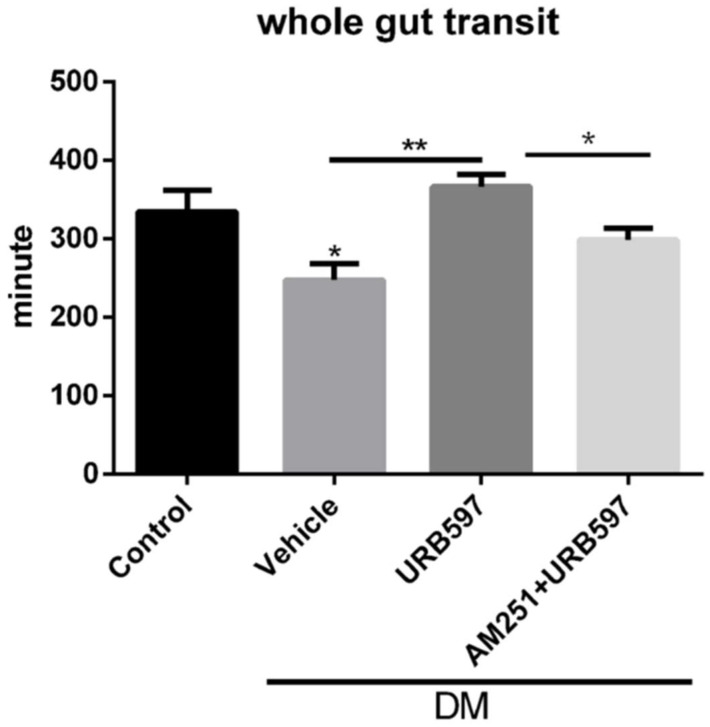
The effects of STZ-induced diabetes and FAAH inhibitor (URB597) on whole gut transit time measured by Evans Blue marker in the mouse. The diabetic mice had a faster transit (* *p ≤* 0.05 vs. control); this effect was normalized with URB597 (5 mg/kg, IP) (** *p ≤* 0.01 vs. vehicle treated diabetic mice). AM251 (0.5 mg/kg, IP) reversed the effects of URB597 on the transit time. (*n* = 5 per group; one-way ANOVA F (3, 28) = 5.039; *p ≤* 0.01).

Based on our observation that diabetic animals had increased fecal output as compared with the controls and that this increased fecal output was lowered by the treatment with URB597, we evaluated the changes in the whole gut transit time, which was assessed by Evans Blue marker. In the STZ-treated diabetic mice, the whole gut transit time was significantly shorter as compared with the vehicle-treated non-diabetic mice (T1D mice 258.84 ± 80.5 min versus control mice 334.28 ± 87.4 min, *p* ≤ 0.05), suggesting a faster gut transit in these animals (Figure 2). Inhibiting FAAH with URB597 at a dose of 5 mg/kg increased the transit time in the diabetic mice (366.41 ± 41.8 min, Figure 2), possibly through an augmentation of the endocannabinoid response. The effects of URB597 on fecal output was eliminated with AM251 at a dose of 0.5 mg/kg (298.33 ± 25.73 min; (*p* ≤ 0.05), suggesting a CB1-mediated mechanism for URB597 in controlling transit time in the diabetic mice (Figure 1). Similar to fecal output experiments, URB597 and AM251 did not change transit time in the control non-diabetic mice (data not shown).

## 4. Discussion

In this study, we examined the influence of gastric dysmotility in STZ-induced type 1 diabetes on the whole gut transit time and the pellet count of fecal matter. The overall aim of the current study was to examine the contribution of the endocannabinoid system in the alteration of gastric emptying in T1D mice. First, the results of this study demonstrate that there was a change in gastrointestinal motion with a significant acceleration in the transit time in diabetes. STZ-injected diabetic animals in the study showed a significant increase in blood glucose, while the blood glucose levels remained steady in the control and the vehicle-treated groups. The induction of diabetes was also associated with no weight gain in diabetic group, whereas the control and vehicle-treated mice gained weight as part of their natural growth. It is well known that diabetes affects gut motility, which affects the whole gut transit time leading to delayed or accelerated gastric emptying rate [28,29]. Decreased sympathetic inhibition can cause diabetic gastric hypermotility; neuropathy due to irregular internal and external anal sphincter function can also induce fecal incontinence [30,31]. A remedy for diabetic fecal incontinence is predominantly observational and focused towards symptomatic relief, improving diet and blood glucose control, and management of any causal effects [31].

**Figure 3:**
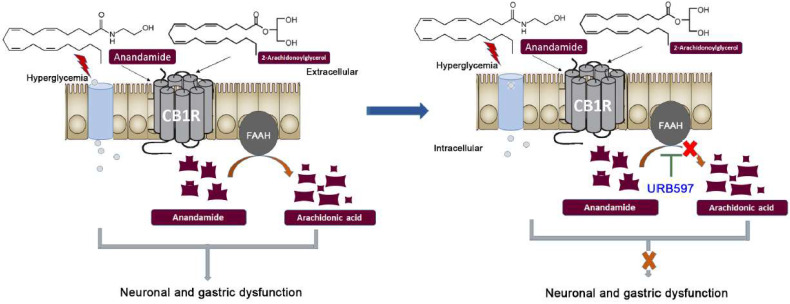
This schematic presentation depicts that natural endocannabinoid anandamide activates the receptors CB1 in the membrane of the ileum and distal colon. The enzyme FAAH breaks down anandamide immediately after use, whereas this study shows that inhibiting FAAH with URB597 results in significant improvement suggesting a CB1-mediated mechanism.

Increased fecal output in diabetic animals suggests neuronal dysfunction in the sympathetic nerves as well as changes in gut permeability. Overall, the results indicate that increased fecal pellet count is due to increased fecal mass and is not primarily restricted to any oversecretion or gut permeability change which may be observed after STZ. Whether this increase in fecal output was due to an increase in food intake in STZ-treated mice needs further investigation, however, it was confirmed that this effect is partly due to the endocannabinoid system, since the effect of increased number of fecal pellets was blocked by the treatment with URB597, a FAAH inhibitor (Figure 3). The effect of URB597 was not reversed by the treatment with CB1 antagonist, AM251. This suggests that increased fecal output may not be mediated by the CB1 receptor. Treatment with AM251 (0.5 mg/kg) [24] alone did not change fecal pellet counts and fecal weights in the control and diabetic mice (preliminary studies, data shown in Supplementary Figure S3). Wrzos et. al reported that STZ-induced T1D rats displayed a reduced number of myenteric neurons [32]. Dysfunction in the absorption capacity of the lower GI tract could also contribute to the incidence of wet stools [8]. Damage to the vagus nerve in STZ-induced T1D animals has been shown to lead to fast entry into the small intestine, ultimately leading to faster bowel movement and wet stools [33].

The effect of URB597 on fecal output was effective for the first 30 min, and then we did not observe any changes, possibly due to a decrease in the baseline fecal material within first 30 min. The effect of FAAH inhibitor on fecal weight was partially reversed by AM251. The proportionally similar changes in both dry and wet fecal material weight suggest that all these changes are mainly in the setting on GI motility rather than the secretary or permeability related mechanisms. This also suggests potential induction of the endocannabinoid enzymes as well as their expression levels. Analogous observations have been documented in previous studies where authors have also documented that inflammation was significantly reduced in the presence of URB597 [27,34]. In STZ-treated diabetic mice, the whole gut transit time was significantly shorter as compared with vehicle-treated non-diabetic mice, suggesting a faster gut transit in these animals (Figure 2). Inhibiting FAAH with URB597 enhanced the transit time in the diabetic mice, which points towards the possibility of increased response of the endocannabinoid system. The effect of URB597 on fecal output was moderately decreased after AM251 treatment, suggesting a CB1-mediated mechanism for URB597 in controlling transit time in diabetic mice. In the current study, we demonstrated that changes in the FAAH inhibitor on the total fecal pellet and the dry weight of the fecal matter were not mediated through the CB1 receptor, as AM251 treatment did not affect these assessments. Therefore, for total fecal output study, whether URB597 induces a CB2-mediated response or its effect is through the induction of other fatty palmitoylethanolamide (PEA) and oleoylethanolamide (OEA) which work through non-CB receptors, needs further investigation. Conversely, we assessed the whole gut transit time by Evans Blue marker to measure gastric motility in a freely moving animal and demonstrated that, in diabetic mice, the whole gut transit time was significantly shorter as compared with vehicle-treated non-diabetic mice. Inhibiting FAAH with URB597 enhanced the transit time in the diabetic mice, whereas the effect of URB597 on the transit time was partially reduced with AM251 (0.5 mg/kg), suggesting a CB1-mediated mechanism for URB597 in controlling transit time in the diabetic mice. We did not identify any influences of the CB1 receptor antagonists on total fecal count or the dry weight. This lack of effect on fluid absorption suggests that the physiological role of the endocannabinoid system may be limited to activation of CB1 receptors which regulate GI motility and is not an over-all contribution of CB1 receptors which are found throughout the enteric nervous system. The current study shows that inhibition of FAAH may present as a new therapeutic approach to the treatment of dysfunctional intestinal motility in diabetes. The commonly used cannabinoids in GI disorders have mostly focused on their property as antiemetic and motility drugs. The scope of this study is limited. Therefore, further investigations with specific cannabinoid CB1 receptor agonists and antagonists will be beneficial to reveal the precise role of these compounds on a spectrum of gastrointestinal disorders including intestinal dysmotility and gastroparesis in diabetic patients.

## Data Availability

All data generated or analyzed during the current study are included in this published article.

## Supplementary Materials

The following supporting information can be downloaded at: https://www.mdpi.com/article/10.3390/vetsci9070364/s1, Figure S1: (a) Random glucose test showing significant difference in average glucose measures in control versus diabetic groups. After STZ injections (Day0). (b) STZ animals didn’t gain any weight for the entire span of the study; Figure S2: No difference in total fecal count in control+vehicle versus control+URB597 treated animals; Figure S3: (a) No difference in total fecal pellet count in control+AM251 versus STZ+AM251 treated animals. (b) No difference in wet fecal pellet weight in control+AM251 versus STZ+AM251 treated animals.

## References

[1] Maisey A. (2016). A Practical Approach to Gastrointestinal Complications of Diabetes. Diabetes Ther..

[2] Bytzer P., Talley N.J., Leemon M., Young L.J., Jones M.P., Horowitz M. (2001). Prevalence of gastrointestinal symptoms associated with diabetes mellitus: A population-based survey of 15,000 adults. Arch. Intern. Med..

[3] Krishnan B., Babu S., Walker J., Walker A.B., Pappachan J.M. (2013). Gastrointestinal complications of diabetes mellitus. World J. Diabetes.

[4] Keller J., Layer P. (2009). Intestinal and anorectal motility and functional disorders. Best Pract. Res. Clin. Gastroenterol..

[5] You S., Anitha M., de Souza S.M., Jia D., Lu X., Kozlowski M., Olson D.E., Srinivasan S., Thule P.M. (2015). Hepatic insulin gene therapy prevents diabetic enteropathy in STZ-treated CD-1 mice. Mol. Ther.-Methods Clin. Dev..

[6] Phillips L.K., Rayner C.K., Jones K.L., Horowitz M. (2006). An update on autonomic neuropathy affecting the gastrointestinal tract. Curr. Diabetes Rep..

[7] Wouters M.M., Vicario M., Santos J. (2016). The role of mast cells in functional GI disorders. Gut.

[8] Spiller R. (2006). Role of motility in chronic diarrhoea. Neurogastroenterol. Motil..

[9] Gruden G., Barutta F., Kunos G., Pacher P. (2016). Role of the endocannabinoid system in diabetes and diabetic complications. Br. J. Pharm..

[10] Fraser R.J., Horowitz M., Maddox A.F., Harding P.E., Chatterton B.E., Dent J. (1990). Hyperglycaemia slows gastric emptying in type 1 (insulin-dependent) diabetes mellitus. Diabetologia.

[11] Bult H., Boeckxstaens G.E., Pelckmans P.A., Jordaens F.H., Van Maercke Y.M., Herman A.G. (1990). Nitric oxide as an inhibitory non-adrenergic non-cholinergic neurotransmitter. Nature.

[12] Schmidt R.E., Plurad S.B., Modert C.W. (1983). Experimental diabetic autonomic neuropathy characterization in streptozotocin-diabetic Sprague-Dawley rats. Lab. Investig..

[13] Tougas G., Hunt R.H., Fitzpatrick D., Upton A.R. (1992). Evidence of impaired afferent vagal function in patients with diabetes gastroparesis. Pacing Clin. Electrophysiol..

[14] DiPatrizio N.V. (2016). Endocannabinoids in the Gut. Cannabis Cannabinoid Res..

[15] Aviello G., Romano B., Izzo A.A. (2008). Cannabinoids and gastrointestinal motility: Animal and human studies. Eur. Rev. Med. Pharmacol. Sci..

[16] Izzo A.A., Camilleri M. (2008). Emerging role of cannabinoids in gastrointestinal and liver diseases: Basic and clinical aspects. Gut.

[17] Pertwee R.G., Ross R.A. (2002). Cannabinoid receptors and their ligands. Prostaglandins Leukot. Essent. Fat. Acids.

[18] Abalo R., Vera G., Lopez-Perez A.E., Martinez-Villaluenga M., Martin-Fontelles M.I. (2012). The gastrointestinal pharmacology of cannabinoids: Focus on motility. Pharmacology.

[19] Hornby P.J., Prouty S.M. (2004). Involvement of cannabinoid receptors in gut motility and visceral perception. Br. J. Pharm..

[20] Howlett A.C., Blume L.C., Dalton G.D. (2010). CB(1) cannabinoid receptors and their associated proteins. Curr. Med. Chem..

[21] Izzo A.A., Fezza F., Capasso R., Bisogno T., Pinto L., Iuvone T., Esposito G., Mascolo N., Di Marzo V., Capasso F. (2001). Cannabinoid CB1-receptor mediated regulation of gastrointestinal motility in mice in a model of intestinal inflammation. Br. J. Pharm..

[22] Pertwee R.G. (2001). Cannabinoids and the gastrointestinal tract. Gut.

[23] Nasser Y., Bashashati M., Andrews C.N. (2014). Toward modulation of the endocannabinoid system for treatment of gastrointestinal disease: FAAHster but not “higher”. Neurogastroenterol. Motil..

[24] Storr M.A., Bashashati M., Hirota C., Vemuri V.K., Keenan C.M., Duncan M., Lutz B., Mackie K., Makriyannis A., Macnaughton W.K. (2010). Differential effects of CB(1) neutral antagonists and inverse agonists on gastrointestinal motility in mice. Neurogastroenterol. Motil..

[25] Chattopadhyay M., Walter C., Mata M., Fink D.J. (2008). Neuroprotective effect of herpes simplex virus-mediated gene transfer of erythropoietin in hyperglycemic dorsal root ganglion neurons. Brain.

[26] Bashashati M., Nasser Y., Keenan C.M., Ho W., Piscitelli F., Nalli M., Mackie K., Storr M.A., Di Marzo V., Sharkey K.A. (2015). Inhibiting endocannabinoid biosynthesis: A novel approach to the treatment of constipation. Br. J. Pharm..

[27] Bashashati M., Storr M.A., Nikas S.P., Wood J.T., Godlewski G., Liu J., Ho W., Keenan C.M., Zhang H., Alapafuja S.O. (2012). Inhibiting fatty acid amide hydrolase normalizes endotoxin-induced enhanced gastrointestinal motility in mice. Br. J. Pharm..

[28] Lupoli R., Creanza A., Griffo E., Nardone G., Rocco A., Bozzetto L., Annuzzi G., Riccardi G., Capaldo B. (2018). Gastric Emptying Impacts the Timing of Meal Glucose Peak in Subjects With Uncomplicated Type 1 Diabetes. J. Clin. Endocrinol. Metab..

[29] Perano S.J., Rayner C.K., Kritas S., Horowitz M., Donaghue K., Mpundu-Kaambwa C., Giles L., Couper J.J. (2015). Gastric Emptying Is More Rapid in Adolescents With Type 1 Diabetes and Impacts on Postprandial Glycemia. J. Clin. Endocrinol. Metab..

[30] Rayner C.K., Samsom M., Jones K.L., Horowitz M. (2001). Relationships of upper gastrointestinal motor and sensory function with glycemic control. Diabetes Care.

[31] Ebert E.C. (2005). Gastrointestinal complications of diabetes mellitus. Dis. Mon..

[32] Wrzos H.F., Cruz A., Polavarapu R., Shearer D., Ouyang A. (1997). Nitric oxide synthase (NOS) expression in the myenteric plexus of streptozotocin-diabetic rats. Dig. Dis. Sci..

[33] Kuznik E., Dudkowiak R., Adamiec R., Poniewierka E. (2020). Diabetic autonomic neuropathy of the gastrointestinal tract. Prz. Gastroenterol..

[34] Storr M.A., Keenan C.M., Emmerdinger D., Zhang H., Yuce B., Sibaev A., Massa F., Buckley N.E., Lutz B., Goke B. (2008). Targeting endocannabinoid degradation protects against experimental colitis in mice: Involvement of CB1 and CB2 receptors. J. Mol. Med..

