# Supplementary material for: Inhibiting Fatty Acid Amide Hydrolase Ameliorates Enteropathy in Diabetic Mice: A Cannabinoid 1 Receptor Mediated Mechanism
Source: Vet Sci. 2022 Jul 16;9(7):364. doi: 10.3390/vetsci9070364 (PMC9319435; doi:10.3390/vetsci9070364)
Supplement: Supplementary file 1 [file vetsci-09-00364-s001.zip › vetsci-1694064-supplementary.pdf]

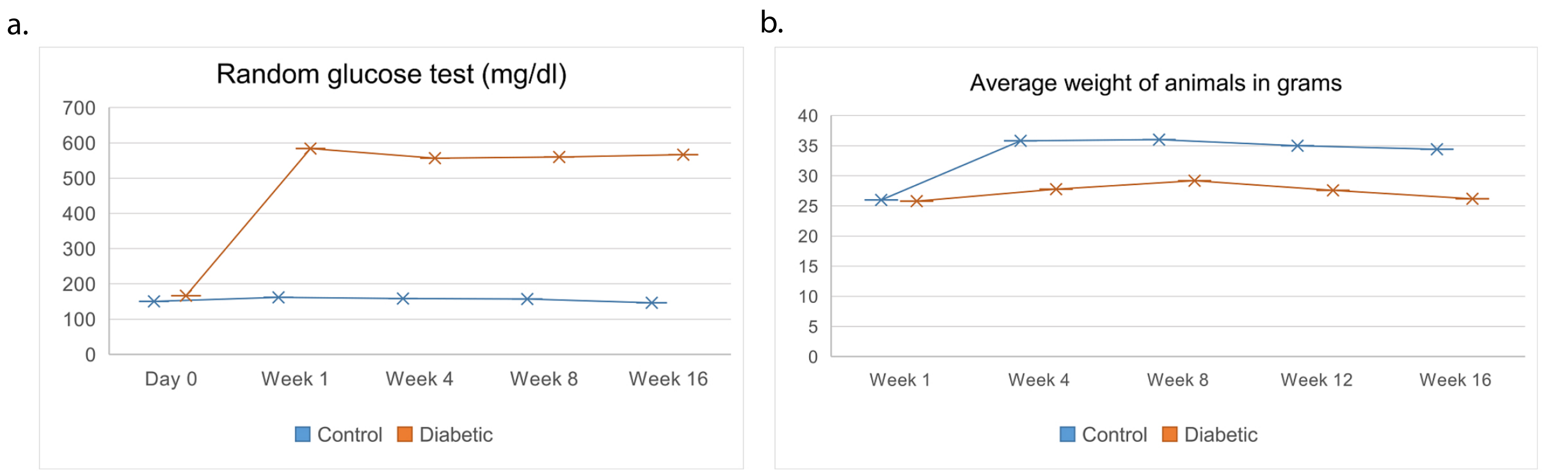

Figure S1. (a) Random glucose test showing significant difference in average glucose measures in control versus diabetic groups after STZ injections (Day 0). (b) STZ animals didn't gain any weight for the entire span of the study.

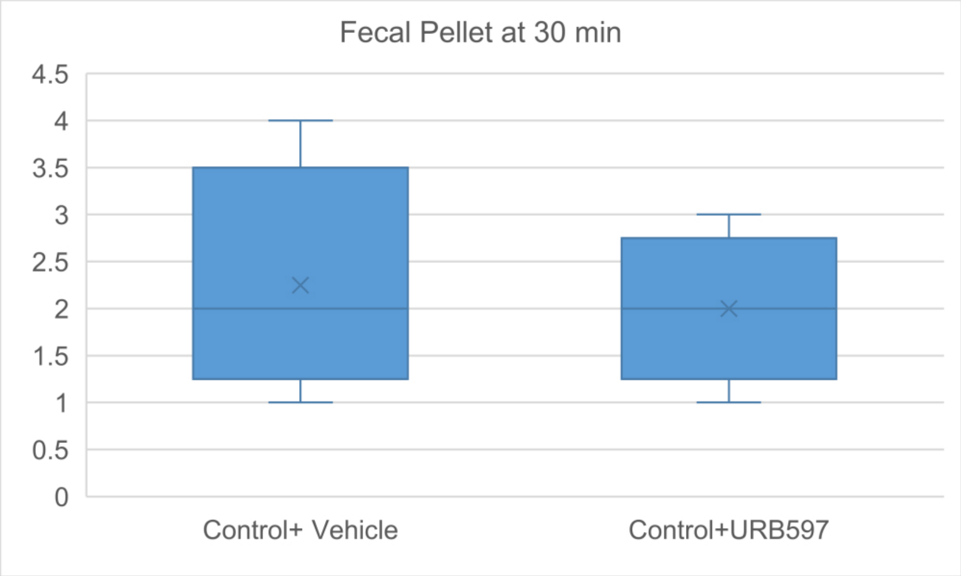

Figure S2. No difference in total fecal count in control+vehicle versus control+URB597 treated animals.

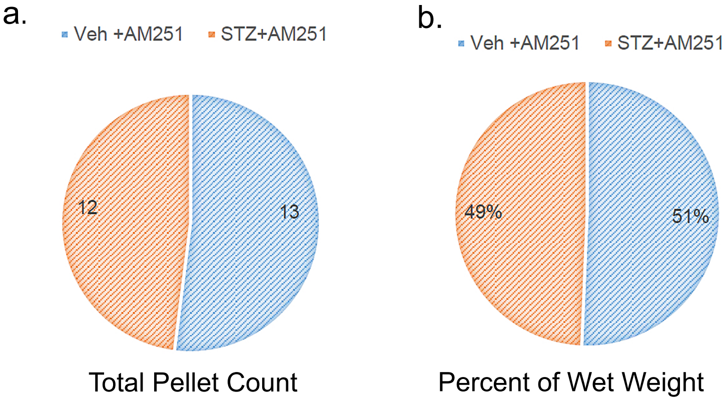

Figure S3. (a) No difference in total fecal pellet count in control+AM251 versus STZ+AM251 treated animals.
(b) No difference in wet fecal pellet weight in control+AM251 versus STZ+AM251 treated animals.
